# Autistic traits associated with dichotomic thinking mediated by intolerance of uncertainty

**DOI:** 10.1038/s41598-023-41164-8

**Published:** 2023-08-28

**Authors:** Noi Suzuki, Masahiro Hirai

**Affiliations:** 1 Department of Cognitive and Psychological Sciences, Graduate School of Informatics, Nagoya University, Furo-cho, Chikusa-ku, Nagoya, 464-8601 Japan; 2 Department of Pediatrics, Jichi Medical University, 3311-1 Yakushiji, Shimotsuke, Tochigi 329-0392 Japan

**Keywords:** Psychology, Human behaviour

## Abstract

A recent cognitive model suggests that autistic individuals may experience dichotomous thinking patterns mediated by intolerance of uncertainty; however, empirical evidence to support this model is lacking. This study aimed to identify the relationships between autistic traits, intolerance of uncertainty, and dichotomous thinking using the Autism Spectrum Quotient, Short Intolerance of Uncertainty Scale, and the Dichotomous Thinking Inventory. We collected data from non-clinical university students (*N* = 151; pilot study) and general adults (*N* = 500; main study) and analyzed the results using structural equation modeling. Both studies showed a significant indirect effect of autistic traits on dichotomous thinking mediated by intolerance of uncertainty. Moreover, the results indicated that intolerance of uncertainty was significantly and positively associated with Autism Spectrum Quotient and Dichotomous Thinking Inventory scores. Conversely, there was a significant negative direct association between Autism Spectrum Quotient and Dichotomous Thinking Inventory scores. This is the first study to demonstrate that autistic traits can result in dichotomous thinking through intolerance of uncertainty. These findings provide insight into the cognitive patterns of autistic individuals.

## Introduction

Autism spectrum disorder (ASD) is a neurodevelopmental condition characterized by clinical heterogeneity, which affects approximately 1 in 36 individuals^1^. ASD is diagnosed based on social communication difficulties, restricted interests, and atypical sensory processing^2^. The genetic background of autistic individuals is generally heterogeneous^3^. Moreover, atypical perceptual and cognitive styles, such as dominant local processing^4–6^, cognitive inflexibility^7^, and intolerance of uncertainty (IU)^8^, have been reported in autistic individuals.

In addition to atypical cognitive profiles, autistic individuals tend to exhibit a unique thinking pattern characterized as “dichotomous thinking”^9^. This is a form of cognitive distortion wherein an individual perceives things as binary––either good or bad, with no gray area or middle ground. This often involves oversimplifying complex issues and ignoring or minimizing nuances or complexities^10^. Dichotomous thinking has several advantages, such as facilitating quick comprehension and decision-making^11^. Although there are anecdotal descriptions of rigid/dichotomous thinking in autistic individuals^12–14^, theoretical and quantitative studies are limited. A recent review study on cognitive rigidity demonstrated that rigidity might include fixed/restricted/special interests, insistence on sameness and routines/rituals, IU, black-and-white mentality, strict adherence to rules, weak central coherence, and task-switching^14^. To our knowledge, there has been no systematic quantitative study on dichotomous thinking in autistic individuals. A previous study did not directly examine dichotomous thinking in autistic individuals. Rather, it investigated the efficacy of a 1-day training for psychological therapists in cognitive behavioral therapy for autistic children and found it was effective. Moreover, the attending therapists were asked open-ended questions like, “Have you encountered any particular issues or challenges in working as a psychological therapist with people with ASD?” Approximately 40% of the therapists reported that autistic individuals exhibit rigidity or dichotomous thinking, which makes successful treatment challenging^9^.

IU is a psychological construct referring to a personal trait of seeking sufficient information to predict an unpredictable event and reacting negatively to unexpected or unknown events^15^. The uncertainty tolerance model was initially developed with reference to generalized anxiety disorder, characterized by excessive and uncontrollable worry^16, 17^. Recent studies have demonstrated that autistic individuals exhibit stronger IU than their typically developed peers^8, 18–21^. Individuals with IU consider it unacceptable that a negative event may occur, however small the probability of its occurrence^22^. This differs from the similar concept of intolerance of ambiguity^23, 24^. Recent studies have shown that IU can result in anxiety in autistic individuals^8, 18^. Moreover, a recent meta-analysis has confirmed that IU is involved in anxiety^25^.

However, it remains unclear how atypical perceptual and cognitive profiles result in biased thinking patterns, such as dichotomous thinking, in autistic individuals. A recent theoretical model proposed by Stark et al. argues that atypical cognitive cascades cause the enhanced anxiety levels frequently observed in autistic individuals^26^. According to this model, autistic individuals may struggle with IU partly because of difficulty making top-down predictions, resulting in dichotomous thinking patterns. Furthermore, IU and dichotomous thinking can interact with anxiety symptoms in autistic individuals^26^. Dichotomous thinking might emerge because of increasing predictability following affective discomfort related to IU and the resulting cognitive-behavioral drive to acquire predictability^26^.

The model proposed in the study^26^ could explain the relationship between IU and dichotomous thinking in autistic individuals; however, this association is hypothetical. Therefore, it is necessary to test whether the cognitive cascade in the proposed model is plausible. This study tested whether autistic traits are related to the tendency toward dichotomous thinking, which is mediated by the tendency toward IU in non-clinical populations. We conducted a survey with a non-clinical Japanese population using three questionnaires that characterize autistic traits, IU, and dichotomous thinking: Adult Autism Spectrum Quotient (AQ)^27^, Japanese version of the Short IU Scale (SIUS)^28^ originally developed by Carleton et al.^29^, and Dichotomous Thinking Inventory (DTI)^11^. We hypothesized that if the model proposed by Stark et al.^26^ is plausible, we would observe a significant indirect effect of autistic traits on dichotomous thinking mediated by IU.

## Pilot study

### Results and discussion

Internal consistency (Cronbach’s α) was reasonable for AQ, IU, and DTI (0.84, 0.85, and 0.87). Descriptive statistics and Pearson’s correlations for all variables are reported in Table 1.

**Table 1:** Pearson’s correlation coefficient results in the pilot study.

	Measure	*M* (*SD*)	1	2	3	4	5	6	7	8	9
1	AQ Social skill	4.68 (2.89)									
2	AQ Attention switch	5.46 (2.17)	0.44***								
3	AQ Attention-to-detail	4.74 (2.24)	− 0.02	0.27***							
4	AQ Communication	3.83 (2.25)	0.64***	0.53***	0.02						
5	AQ Imagination	3.47 (2.10)	0.50***	0.24**	− 0.02	0.53***					
6	SIUS Prospective anxiety	21.48 (4.52)	0.29***	0.45***	0.25**	0.31***	0.19*				
7	SIUS Inhibitory anxiety	13.00 (4.16)	0.31***	0.40***	0.21**	0.38***	0.20*	0.68***			
8	DTI Preference for dichotomy	17.62 (4.38)	0.02	0.10	0.21**	− 0.04	0.03	0.40***	0.25**		
9	DTI Dichotomous belief	11.70 (4.32)	− 0.01	0.01	0.08	− 0.06	0.04	0.26**	0.19*	0.52***	
10	DTI Profit-and-loss thinking	20.56 (4.14)	0.07	0.22**	0.09	0.05	0.10	0.46***	0.19*	0.60***	0.40***

AQ, Autism Quotient; SIUS, Short Intolerance of Uncertainty Scale; DTI, Dichotomous Thinking Inventory.

*p < 0.05, **p < 0.01, ***p < 0.001.

**Figure 1:**
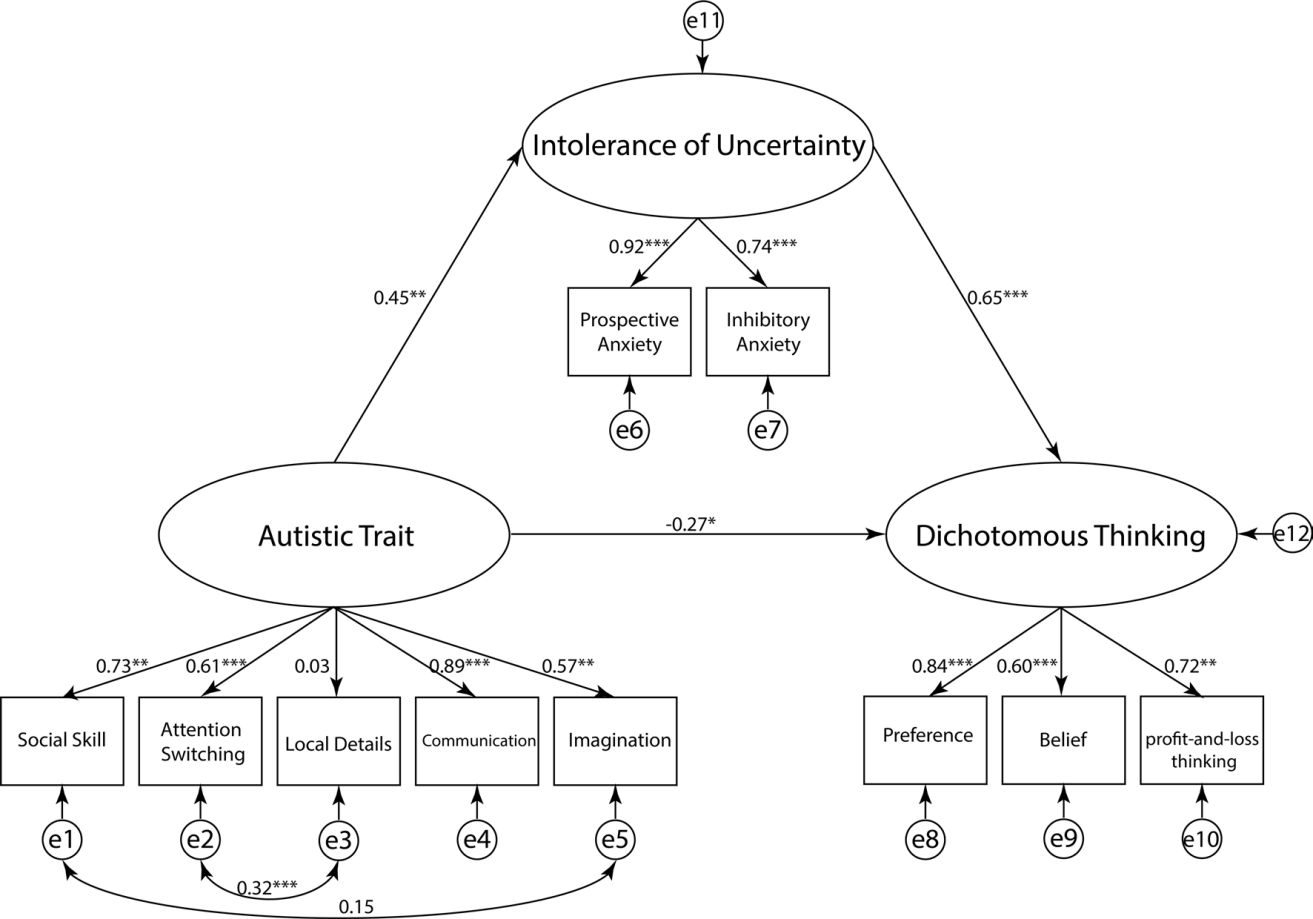
Structural equation model (SEM) for the best-fitting model in the pilot study. The value on each path indicates a standardized partial regression coefficient. *p < 0.05, **p < 0.01, ***p < 0.001.

For the overall model fit indices^30^, the goodness of fit index (GFI) was acceptable (maximum likelihood chi-square [MLχ^2^] (30) = 57.57, *p* = 0.002; MLχ^2^/df = 1.92; GFI = 0.93; adjusted goodness-of-fit index [AGFI] = 0.88; root mean square error of approximation [RMSEA] = 0.078, standardized root mean square residual [SRMSR] = 0.082, comparative fit index [CFI] = 0.94, Akaike information criterion [AIC] = 107.6; Fig. 1). Regarding the direct effects (Table 2), higher autistic traits were associated with higher IU (*b* = 0.46, 95% CI [0.20, 0.65], *p* = 0.001). Moreover, IU was positively associated with dichotomous thinking (*b* = 0.65, 95% CI [0.42, 0.89], *p* < 0.001). In contrast, autistic traits were negatively associated with dichotomous thinking (*b* = − 0.27, 95% CI [− 0.52, − 0.031], *p* = 0.031). Regarding the indirect effects (Table 2), autistic traits were positively associated with DTI scores mediated by IU (*b*  = 0.74, 95% CI [0.31, 1.66], *p* < 0.001). The results indicated a positive association between autistic traits and IU. Moreover, IU was also positively associated with dichotomous thinking. Conversely, autistic traits were negatively associated with dichotomous thinking. This suggests that people with high autistic traits may struggle with IU partly because of difficulty making top–down predictions, resulting in dichotomous thinking patterns.

**Table 2:** Indirect effects and 95% confidence intervals (CIs) for the model in the pilot study.

Model pathways	Estimated	95% CI	
		Lower	Upper
Autistic trait → Intolerance of uncertainty	0.46***	0.20	0.65
Autistic trait → Dichotomous thinking	− 0.27*	− 0.52	− 0.03
Intolerance of uncertainty → Dichotomous thinking	0.65***	0.42	0.89
Autistic trait → Intolerance of uncertainty → Dichotomous thinking	0.74***	0.31	1.70

*p < .05, ***p < .001.

## Main study

The main study had the same aims as the pilot study; however, while the pilot study examined university students, the main study recruited an appropriate number of participants from various occupations. Furthermore, we aimed to replicate the pilot study findings by testing many participants to evaluate the robustness of the initial results.

### Results and discussion

Internal consistency (Cronbach’s α) was reasonable for AQ, IU, and DTI (0.67, 0.88, and 0.91, respectively). Descriptive statistics and Pearson’s correlations for all variables are reported in Table 3.

**Table 3:** Pearson’s correlation coefficient results in the main study.

	Measure	*M* (*SD*)	1	2	3	4	5	6	7	8	9
1	AQ Social skill	5.66 (2.44)									
2	AQ Attention switch	5.54 (1.76)	0.44***								
3	AQ Attention-to-detail	4.89 (1.97)	− 0.15**	− 0.04							
4	AQ Communication	4.66 (1.98)	0.43***	0.43***	0.00						
5	AQ Imagination	4.49 (1.87)	0.34***	0.15**	− 0.13**	0.35***					
6	SIUS Prospective anxiety	20.80 (5.20)	0.25***	0.29***	0.03	0.20***	0.08				
7	SIUS Inhibitory anxiety	14.16 (4.18)	0.27***	0.28***	0.00	0.30***	0.09*	0.70***			
8	DTI Preference for dichotomy	16.94 (4.58)	0.10*	0.12**	0.17**	0.06	0.03	0.47***	0.43***		
9	DTI Dichotomous belief	15.35 (5.07)	0.12**	0.11*	0.13**	0.10*	0.14**	0.37***	0.39***	0.70***	
10	DTI Profit-and-loss thinking	18.60 (4.95)	0.08	0.17**	0.12**	0.03	− 0.04	0.50***	0.44***	0.77***	0.53***

AQ, Autism Quotient; SIUS, Short Intolerance of Uncertainty Scale; DTI, Dichotomous Thinking Inventory.

*p < 0.05, **p < 0.01, ***p < 0.001.

**Figure 2:**
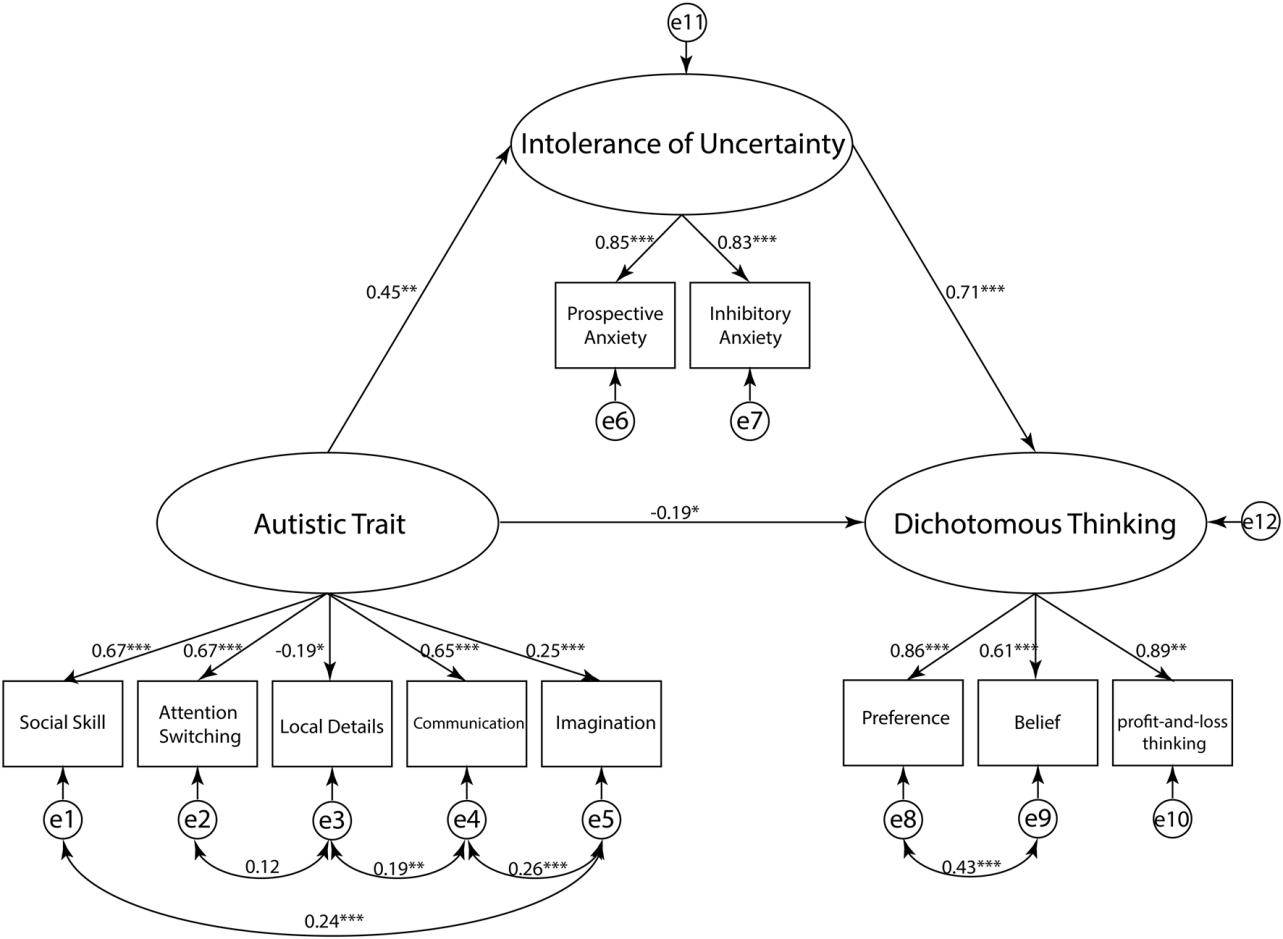
Structural equation model (SEM) for the best-fitting model in the main study. The value on each path indicates a standardized partial regression coefficient. *p < 0.05, **p < 0.01, ***p < 0.001.

For the overall model fit indices^30^, the GFI was acceptable (MLχ^2^ (27) = 80.71, *p* < 0.001; MLχ^2^ /df = 2.99; GFI = 0.97; AGFI = 0.94; RMSEA = 0.063, SRMSR = 0.055, CFI = 0.97, AIC = 136.7; Fig. 2). Regarding the direct effects (Table 4), higher autistic traits were associated with higher IU (*b* = 0.45, 95% CI [0.35, 0.55], *p* = 0.001). Moreover, IU was positively associated with dichotomous thinking (*b* = 0.71, 95% CI [0.58, 0.84], *p* < 0.001). In contrast, autistic traits were negatively associated with dichotomous thinking (*b* = − 0.19, 95% CI [− 0.33, − 0.061], *p* = 0.003). Regarding the indirect effects (Table 4), autistic traits were positively associated with DTI scores mediated by IU (*b* = 2.99, 95% CI [1.68, 6.97], *p* < 0.001).

**Table 4:** Indirect effects and 95% confidence intervals (CIs) for the model in the main study.

Model pathways	Estimated	95% CI	
		Lower	Upper
Autistic trait → Intolerance of uncertainty	0.45***	0.35	0.55
Autistic trait → Dichotomous thinking	− 0.19*	− 0.33	− 0.06
Intolerance of uncertainty → Dichotomous thinking	0.71***	0.58	0.84
Autistic trait → Intolerance of uncertainty → Dichotomous thinking	2.99***	1.68	6.97

*p < .05, *** *p* < .001.

As in the pilot study, the main study revealed a similar tendency in the relationships between autistic traits, IU, and dichotomous thinking. IU was positively associated with autistic traits and dichotomous thinking. In addition, there was a negative association between autistic traits and dichotomous thinking.

## General discussion

The pilot and main studies examined how autistic traits among non-clinical Japanese university students and adults could result in dichotomous thinking through IU. Based on the hypothetical model proposed by Stark et al.^26^, we investigated potential pathways from autistic traits to dichotomous thinking. The structural equation modeling (SEM) analysis revealed that autistic traits measured using AQ produced dichotomous thinking mediated by IU in both the pilot and main studies, concordant with the theoretical model^26^. Furthermore, we found a significant negative association between autistic traits and dichotomous thinking.

The pilot and main studies indicate that higher autistic traits were associated with higher IU in a non-clinical population of university students and general adults. This is consistent with a study that found that autistic children reported higher IU than those without ASD^8^. The relationship between IU and anxiety in autistic individuals was confirmed in a recent meta-analysis^25^. In addition, we demonstrated that autistic traits in non-clinical populations are positively correlated with IU.

Furthermore, we found a significant positive correlation between IU and dichotomous thinking. This is the first study to show that the degree of IU can directly modulate a binary mode of thinking. This association might be explained using a predictive coding framework. As noted by Stark et al.^26^, “One possible link between intolerance of uncertainty and black-and-white thinking in autism is that to circumvent the discomfort of uncertainty and not end up feeling ‘stuck,’ autistic individuals may tend to attribute a binary outcome to uncertain states of truth and thereby end up at a ‘black or white’ outcome that feels certain, thereby reducing their anxiety. Black-and-white thinking may therefore be a safety behavior, conscious or not, used by autistic individuals to reduce uncertainty and associated anxiety in the short term” (p. 576). Therefore, the positive link between autistic traits and dichotomous thinking may reflect a “protective” strategy to reduce anxiety. The lack of computation of prediction errors concerning the surrounding environment could explain a preference for a predictable outcome^31^. Likewise, autistic individuals could develop prior expectations, as in typically developed individuals; however, they experienced difficulties adjusting the prior expectations to new contexts^32^. Similarly, higher-order restricted interests and insistence on similarity in autistic individuals might indicate a strategy to reduce uncertainty related to real-life events and increase the predictability of life^8, 33^.

Contrary to our hypothesis, we found a significant negative association between autistic traits and dichotomous thinking in both studies. This contradicts previous anecdotal descriptions regarding the relationship between autistic traits and rigid thinking^12, 13^ and a recent research^9^. One study indicated that a large proportion of counselors (40%) engaged in the therapeutic treatment of autistic individuals frequently encountered barriers or issues due to rigidity or dichotomous thinking among them^9^. To our knowledge, the quantitative relationship between autistic traits and dichotomous thinking has not been tested. Therefore, it is difficult to presume that autistic traits are significantly associated with dichotomous thinking patterns. However, as outlined above, our results imply that autistic traits cannot simply induce a tendency toward binary thinking; rather, dichotomous thinking might be mediated by IU.

The significant indirect effect across autistic traits and dichotomous thinking mediated by IU could be related to cognitive atypicality, such as cognitive flexibility and difficulties in updating an internal model, in autistic individuals. From a computational perspective, ASD is associated with difficulties in predictive abilities^31^ and updating priors^32^. Furthermore, cognitive inflexibility involves salience detection and attention, working memory, inhibition, and switching^34^, which can enhance IU. In support of this possibility, a recent study demonstrated that cognitive inflexibility is important in the link between ASD symptoms and aggressive or outburst behaviors and has an indirect role in anxiety mediated by IU^35^. However, as we did not directly test the role of cognitive inflexibility and IU in the current study, further research is needed.

Dichotomous thinking has been reported not only in autistic individuals but also in individuals with eating disorders, indicating a potential similarity in cognitive processes between these two groups. The Dichotomous Thinking in Eating Disorders Scale was initially developed in the context of eating disorders^36^. Moreover, studies have demonstrated that individuals with eating disorders resist conventional therapies, similar to autistic individuals^37^. Furthermore, there is a comorbidity of eating disorders in autistic individuals^38, 39^. Hence, the presence of shared cognitive processes between these two populations can be inferred. The mediating role of IU in the relationship between autistic traits and dichotomous thinking observed in this study should be examined among individuals with other disorders.

This study had several limitations. Although it clarified the relationship between autistic traits, IU, and dichotomous thinking in university students and general adults aged 20–22 years, it did not investigate autistic individuals. Further studies on autistic individuals are required to determine the validity of the present model. Second, the age range was limited; therefore, testing developmental trajectories and whether the current findings hold for younger and older populations is necessary. Third, using a questionnaire, which is a subjective measure, we elucidated the relationship between the three variables; however, it is necessary to examine these relationships using objective measures. Fourth, as we conducted a cross-sectional study, not a longitudinal study, it is difficult to determine the causal relationship across components. A longitudinal study should be conducted to explore the causal relationships. Finally, although the model proposed by Stark et al. states that anxiety is associated with IU and dichotomous thinking, we did not test the relationship between these components and anxiety. As IU is likely associated with anxiety^8^, it is thus important to test the links with anxiety in future research.

## Conclusion

In conclusion, this study revealed that IU plays a mediating role in the relationship between autistic traits and dichotomous thinking. Although previous studies have identified the relationship between IU and anxiety, this is the first study to explore the relationship between autistic traits and dichotomous thinking based on the hypothetical model proposed by Stark et al. using SEM analysis of data collected via three questionnaires. Despite the aforementioned limitations, this is an important first step in elucidating the structure of thinking patterns of autistic individuals. Further studies are required to determine the aspects of autistic traits that can induce unique thinking patterns.

## Methods

### Pilot study

#### Participants

A total of 153 students from Nagoya University were recruited through the online Sona system and participated in the experiment. The participants were sent a Qualtrics link (Qualtrics, Provo, UT) and assessed online. Informed consent was obtained from all participants before they responded to the questionnaires. The participants received an Amazon gift card worth 400 Japanese yen for their participation. This study was approved by the Department of Cognitive and Psychological Sciences ethics committee at Nagoya University (NUPSY-2200929-R-01) and was performed per the Declaration of Helsinki. All methods were performed under the relevant guidelines and regulations. Two participants were excluded: one with missing data and another who did not provide gender information. The final sample comprised 151 participants (male = 73, female = 78, age range = 18–27 years, mean ± SD = 21.3 ± 1.68 years).

#### Materials

The participants were assessed using three questionnaires. First, to assess their autistic traits, the Adult AQ^27^ was administered. The AQ is a 50-item questionnaire that identifies autistic traits and comprises five subscales: social skills, attention switching, attention to detail, communication, and imagination. Each item is rated on a 4-point Likert scale ranging from “definitely disagree” to “definitely agree.” One point is allocated for each response that indicates autistic traits (“definitely agree” or “slightly agree”). However, in the case of a reversed item, one point is allocated for responses of “slightly disagree” or “definitely disagree.”

We assessed IU using the Japanese version of the 12-item SIUS^28^ originally developed by Carleton et al.^29^. This version of the SIUS was developed by extracting 12 items from the original SIUS, which comprised 27 items^17^. Items of the SIUS are rated on a 5-point Likert scale, with responses ranging from 1 (not applicable at all) to 5 (very applicable). The instrument comprises two subscales: prospective and inhibitory anxiety. The former indicates fear and anxiety for future events, and the latter denotes uncertainty inhibiting action or experience.

We assessed dichotomous thinking using the DTI^11^, which comprises three subscales: preference for dichotomy, dichotomous belief, and profit-and-loss thinking. Preference for dichotomy denotes that an individual can better understand or feel when dividing things into two parts. Dichotomous belief denotes that complex events can be divided into two distinct types. Profit-and-loss thinking denotes dividing things into two categories with an orientation toward defining each item as a loss (disadvantage) or gain (advantage). Responses to the DTI are rated on a 6-point Likert scale, with responses ranging from 1 (strongly disagree) to 6 (strongly agree). We used the Qualtrics function to randomize each questionnaire.

#### Data analysis

We conducted SEM to test whether autistic traits modulated dichotomous thinking mediated by IU. Descriptive statistics and correlations were analyzed using the Statistical Package for Social Sciences (SPSS) version 29, and R studio^40^. The SEM analysis was conducted using Analysis of Moments Structures (AMOS) version 29. We evaluated the model using several indices, namely the MLχ^2^ test, GFI, AGFI, CFI, normed fit index, RMSEA, SRMSR, and AIC. For the overall model fit indices^30^, either a GFI ≥ 0.93 or an SRMSR ≤ 0.08 indicates an acceptable fit when the sample size exceeds 100. Direct and indirect effects were analyzed using a bootstrapping method with 5000 resamples, and significance was denoted by a p-value less than 0.05.

### Main study

#### Participants

A total of 500 participants (male = 250, female = 250, age range = 20–22 years, mean ± SD = 21.6 ± 0.9 years) were recruited through an online survey (Cross Marketing Inc.). This study was approved by the ethics committee at the Department of Cognitive and Psychological Sciences at Nagoya University (NUPSY-230506-R-01) and was performed per the Declaration of Helsinki. All methods were performed under the relevant guidelines and regulations.

#### Statistical analysis

All questionnaires and analysis procedures were identical to the pilot study.

## Data Availability

The datasets are available from the corresponding author upon reasonable request.
